# Epithelial CXCL1 tracks clinical remission in inflammatory bowel disease in a disease-specific manner

**DOI:** 10.3389/fimmu.2026.1904951

**Published:** 2026-07-28

**Authors:** Sanmi E. Alake, Anil H. Kadam, Traci Jester, Craig L. Maynard, Babajide A. Ojo

**Affiliations:** 1 Department of Pediatrics, Division of Gastroenterology, Hepatology and Nutrition, University of Alabama at Birmingham, Birmingham, AL, United States; 2 Division of Pulmonary, Allergy and Critical Care Medicine, University of Alabama at Birmingham, Birmingham, AL, United States; 3 Gregory Fleming James Cystic Fibrosis Research Center, University of Alabama at Birmingham, Birmingham, AL, United States; 4 Department of Pathology, Division of Molecular and Cellular Pathology, University of Alabama at Birmingham, Birmingham, AL, United States; 5 Department of Medicine, Division of Gastroenterology and Hepatology, University of Alabama at Birmingham, Birmingham, AL, United States

**Keywords:** chemokines, Crohn’s disease, CXCL1, epithelial cells, epithelial progenitor cells, organoids, ulcerative colitis

## Abstract

**Introduction:**

Stem cell-derived organoids are promising platforms for therapeutic screening in inflammatory bowel disease (IBD), but identifying functional organoid readouts with translational utility is challenging. Colon epithelial organoids from patients with ulcerative colitis (UC) overexpress chemokines CXCL1, CXCL11, CCL2, and CCL28, yet whether these epithelial inflammatory signatures correlate with disease activity and treatment response is unclear. This brief report explores whether organoid-retained chemokines correlate with disease activity and therapeutic outcomes.

**Methods:**

We interrogated three bulk and two single-cell transcriptomic datasets from IBD clinical trials encompassing anti-TNFα and anti-integrin therapies to determine whether epithelial chemokines retained in UC organoids are associated with clinical response and distinguish treatment responders from non-responders to biologic therapy across multiple IBD patient cohorts.

**Results:**

In bulk transcriptomic data, CXCL1, CXCL11, and CCL2 were elevated in active UC and normalized only in patients achieving clinical remission, independent of therapy class, with persistent chemokine overexpression in non-responders. Linear regression analysis demonstrated that CXCL1 showed the strongest and most consistent association with Mayo clinical disease activity scores across independent cohorts. Single-cell analysis of epithelial cell types identified CXCL1 as the only chemokine significantly enriched in both enterocytes and LGR5-positive stem cells in UC. CXCL1 expression normalized in remission but remained elevated in non-remission patients, and this pattern was recapitulated in LGR5-high stem cell states. Epithelial CXCL1 expression was not associated with clinical remission in Crohn’s disease.

**Conclusions:**

CXCL1 is a remission-associated epithelial chemokine in UC and may represent a candidate biomarker and functional endpoint for epithelial-directed therapeutic screening using patient-derived organoid models.

## Introduction

More than half of patients with inflammatory bowel disease (IBD) fail to achieve clinical remission on conventional immunotherapies (1). Since epithelial barrier integrity and mucosal healing are essential for sustained remission, identifying epithelial-directed therapeutic targets represents an unmet need. Patient-derived organoids (PDOs) have emerged as physiologically relevant platforms for preclinical drug screening in IBD (2); however, identifying functional endpoints in IBD organoids that are associated with therapeutic response and clinical remission is unclear.

A defining feature of IBD is the chemokine-directed leukocyte recruitment to the intestinal mucosa to propagate inflammation (3). In particular, cryptitis frequently precedes crypt abscess formation and epithelial barrier damage (4, 5). Notably, epithelial organoids from patients with ulcerative colitis (UC) showed autonomous overexpression of chemokines, including CXCL1, CXCL11, CCL2, and CCL28 (6), which coordinate the recruitment of neutrophils, T cells, monocytes, and plasma cells. Thus, these epithelial chemokines may constitute a durable epithelial inflammatory program that may be therapeutically targeted in UC organoids. However, the clinical relevance of these epithelial chemokine signatures has not been fully established.

In this exploratory study, we analyze bulk and single-cell transcriptomic datasets from multiple clinical trials to determine whether epithelial chemokine expression reflects disease activity and therapeutic response in UC and CD.

## Methods

Publicly available transcriptomic datasets from clinical trials of biologic therapies in inflammatory bowel disease were analyzed, including anti-TNF (golimumab- GSE92415, infliximab-GSE73661) and anti-integrin (vedolizumab-GSE73661) cohorts with paired gene expression and clinical outcome data based on reduction in Mayo scores over the duration of the studies (7, 8). Clinical remission and non-remission groups were defined according to the criteria used in the original TAURUS-IBD study (9). For Crohn’s disease, remission was defined by meeting at least two of the following criteria: Harvey-Bradshaw Index (HBI) < 5, Nancy histological index ≤ 1, and absence of macroscopic ulcers on endoscopy. For ulcerative colitis, remission was defined by meeting at least two of the following criteria: Simple Clinical Colitis Activity Index (SCCAI) ≤ 2, Ulcerative Colitis Endoscopic Index of Severity (UCEIS) ≤ 1, and Nancy histological index ≤ 1. Patients who did not meet these remission criteria, or who required treatment escalation including switching from anti-TNF therapy to another advanced therapy or surgery because of refractory disease, were classified as non-remission.

For bulk microarray datasets, data were extracted from GEO and analyzed in R (v.4.4.0). The Robust Multiarray Average method was used to obtain log2 expression values for each gene. log2-normalized probe intensities were analyzed using ANOVA with FDR adjustment. TPM data were analyzed with Mann-Whitney test for two groups, while Kruskal-Wallis test with Dunn’s *post hoc* was used for more than 2 groups. Statistical analyses were done with GraphPad Prism (v7.04). The expression of our chemokines of interest (CXCL1, CXCL11, CCL2, and CCL28) and Mayo scores in responders and non-responders were extracted for further analyses. The relationship between Mayo scores and chemokine expression levels was assessed using simple linear regression implemented in R using the lm() function from the base *stats* package.

To evaluate the biomarker potential of chemokines, receiver operating characteristic (ROC) analyses were performed to evaluate the ability of chemokine expression to discriminate treatment responders from non-responders across vedolizumab, golimumab, and infliximab cohorts. Analyses were conducted separately for each treatment cohort using binary response status as the outcome variable. ROC curves were generated for baseline expression, post-treatment expression, and normalization (Δchemokine, calculated as baseline expression minus post-treatment expression). The area under the curve (AUC) and corresponding 95% confidence intervals (CI) were calculated using the DeLong method (10) Optimal classification thresholds were determined using the Youden index, with sensitivity and specificity reported at the optimal cutoff. Differences in discriminatory performance between baseline expression and Δ were assessed using bootstrap tests for correlated ROC curves. All analyses were performed in R using the *pROC* package, with a two-sided *P* value < 0.05 considered statistically significant.

To view the expression pattern of chemokines of interest in the epithelium of IBD tissues, we analyzed the public single-cell RNA-seq data obtained from 18 UC patients and 12 healthy controls (11), single cell portal accession number: SCP259). Matrices and metadata for 123006 colon epithelial cells were downloaded from the UCSC Cell Browser (https://human-colon.cells.ucsc.edu), normalized with Seurat (v5.3.1), and visualized using the ggplot2 (v4.0.0) package in R.

To determine the cell type-specific expression and resolution of epithelial chemokines following biologic therapy, processed single-cell transcriptomics data from the TAURUS-IBD study (9) was downloaded from Zenodo (https://doi.org/10.5281/zenodo.13768607), analyzed using Scanpy (v1.9.8) in Python (v3.9.23). UMAP (Uniform Manifold Approximation and Projection) of the available epithelial cell types were visualized with Matplotlib (v3.9.2). For the unsupervised sub-clustering of epithelial stem cells, LGR5+ intestinal stem cells (n=33,568) from the TAURUS_IBD study were isolated and Principal component analysis (PCA) was performed on highly variable genes to obtain 50 principal components. Batch effects were assessed using Chi-squared tests with Cramér’s V statistic and batch correction was applied using Harmony (11) with default parameters. Post-batch correction, k-nearest neighbor graphs (k=15, Euclidean distance) were computed on harmonized PCA coordinates using scikit-learn’s ball_tree algorithm (12). UMAP was applied to the k-NN graph with parameters (min_dist=0.1, spread=1.0, random_state=42) to obtain coordinates for visualization. Using Louvain clustering (resolution=0.2) on harmonized PCA neighbors, cells in the LGR5+ stem cluster were stratified into three stemness states based on LGR5 expression: Stem-hi (high LGR5), Stem-mid (medium LGR5), and Stem-low (low LGR5). This classification was further validated using other established stem cell markers (ASCL2, SMOC2). For quantitative fold-change mapping across individual epithelial and stem-specific clusters, a log-transformation floor of c = 0.1 (log_2_(X + 0.1)) was implemented. Chemokine expression (CXCL1, CXCL11, CCL2, CCL28) was quantified across cell types, disease status (UC, CD, healthy), remission status (Remission vs Non-Remission), and treatment timepoint (Pre vs Post anti-TNF therapy). Differential expression analysis used Mann-Whitney U tests with batch included as a statistical covariate via linear regression (expression ~ disease + batch). False discovery rate (FDR) correction was applied using the Benjamini-Hochberg method. Findings were considered significant at FDR-adjusted p<0.01 with |Log_2_ FC| ≥ 0.5.

## Results

**Figure 1:**
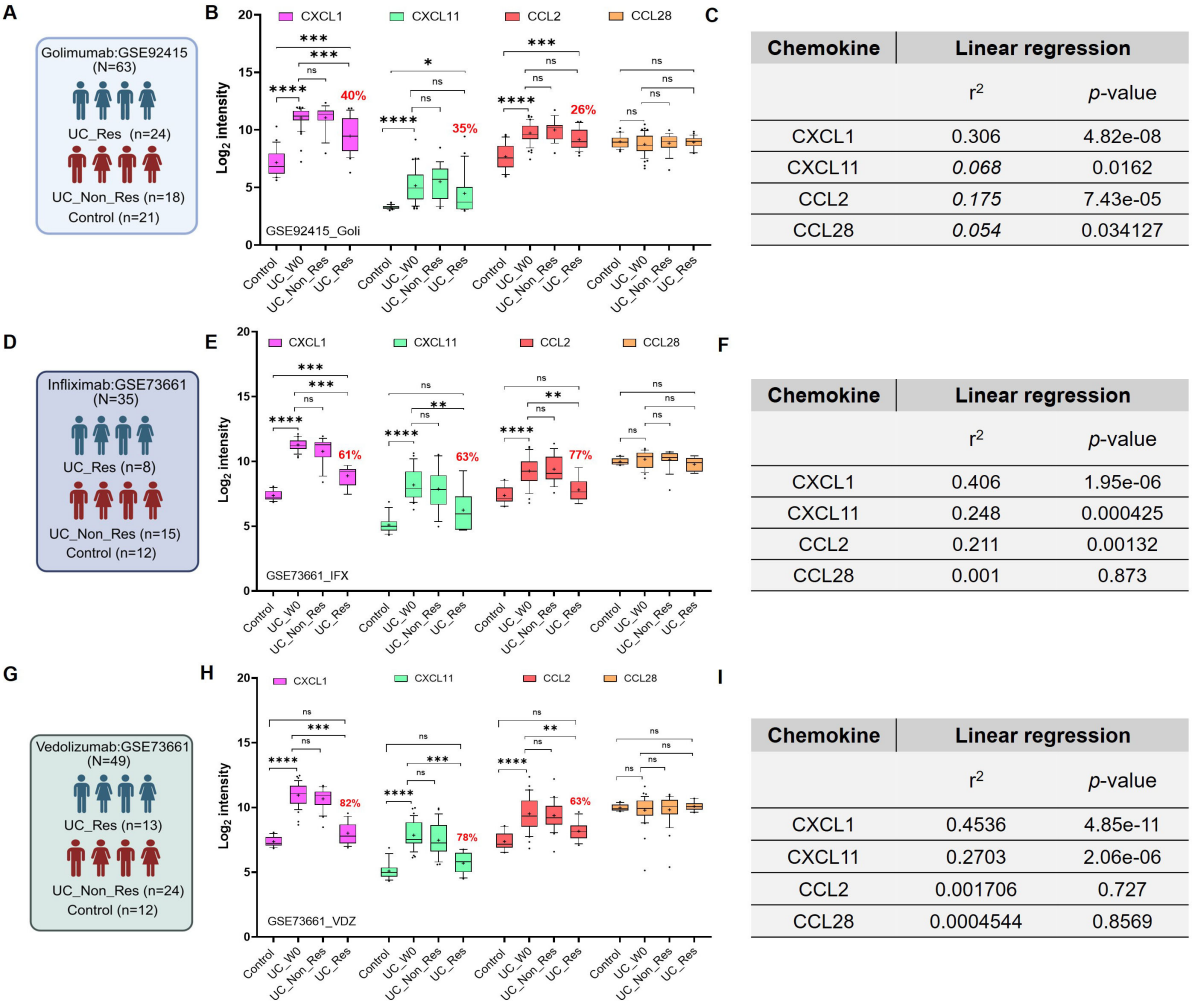
Bulk transcriptomics reveal chemokine expression patterns in UC colonic mucosa across three cohorts before and after biologic treatments. Schematic of the patient cohort are shown in **(A, D)** and **(G)** Box plots **(B, E, H)** showing Log2 expression intensity of CXCL1, CXCL11, CCL2, and CCL28 in healthy controls (Control), UC patients at baseline prior to treatment (UC_W0), UC non-responders (UC_Non_Res), and UC responders (UC_Res) treated with golimumab **(B)**, infliximab **(E)**, or vedolizumab **(H)**. Percentages indicate the efficacy of the therapy on chemokine expression in UC. *p<0.05, **p<0.001, ***p<0.0001; ns = not significant. **(C, F, I)** Linear regression analysis of CXCL1, CXCL11, CCL2, CCL28, and Mayo clinical disease activity score in the golimumab **(C)**, infliximab **(F)**, and vedolizumab **(I)** cohorts. Schematics in **(A, D, G)** were prepared in BioRender.

To investigate whether chemokines elevated in UC organoids exhibit a similar expression pattern and resolve in clinical remission in IBD, we analyzed publicly available microarray datasets from three independent UC patient cohorts treated with distinct biologic agents. Across multiple therapeutic cohorts that analyzed bulk tissue gene expression patterns, the chemokines CXCL1, CXCL11, and CCL2 were consistently elevated at baseline and demonstrated a striking pattern following treatment (**Figures 1B, E, H**). Normalization of chemokine expression occurred only in patients who achieved clinical remission, while non-responders retained elevated expression regardless of therapy type or duration. This pattern was observed across anti-TNF and anti-integrin therapies, suggesting that normalization of epithelial chemokines reflects a shared endpoint of effective treatment rather than a therapy-specific effect. In contrast, CCL28 expression remained relatively stable across disease states and treatment outcomes, indicating that not all chemokines are dynamically regulated in relation to clinical response (**Figures 1B, E, H**). Whereas linear regression analysis confirmed that CXCL1 and CXCL11 expression were significantly associated with Mayo clinical disease activity scores across datasets, CXCL1 showed the strongest relationship (r² of 0.306 to 0.4536 across all cohorts, **Figures 1C, F, I**). CCL2 showed significant association in the golimumab and infliximab cohorts, whereas CCL28 generally lacks or shows weak associations across cohorts (**Figures 1C, F, I**).

Since CXCL1 showed the strongest relationship in our regression analysis, we evaluated its biomarker potential by performing ROC analyses across vedolizumab, golimumab, and infliximab cohorts. CXCL1 normalization (ΔCXCL1; baseline minus post – treatment expression) distinguished responders from non-responders across therapies (AUC range 0.74 – 0.97), with particularly strong performance in vedolizumab (AUC = 0.974) and infliximab (AUC = 0.867) cohorts (**Supplementary Table 1**, **Supplementary Figures 1A–C**). Notably, ΔCXCL1 significantly outperformed baseline CXCL1 in the vedolizumab cohort (P = 0.0008, **Supplementary Table 1**), supporting the concept that CXCL1 resolution rather than baseline expression is more closely associated with therapeutic remission.

**Figure 2:**
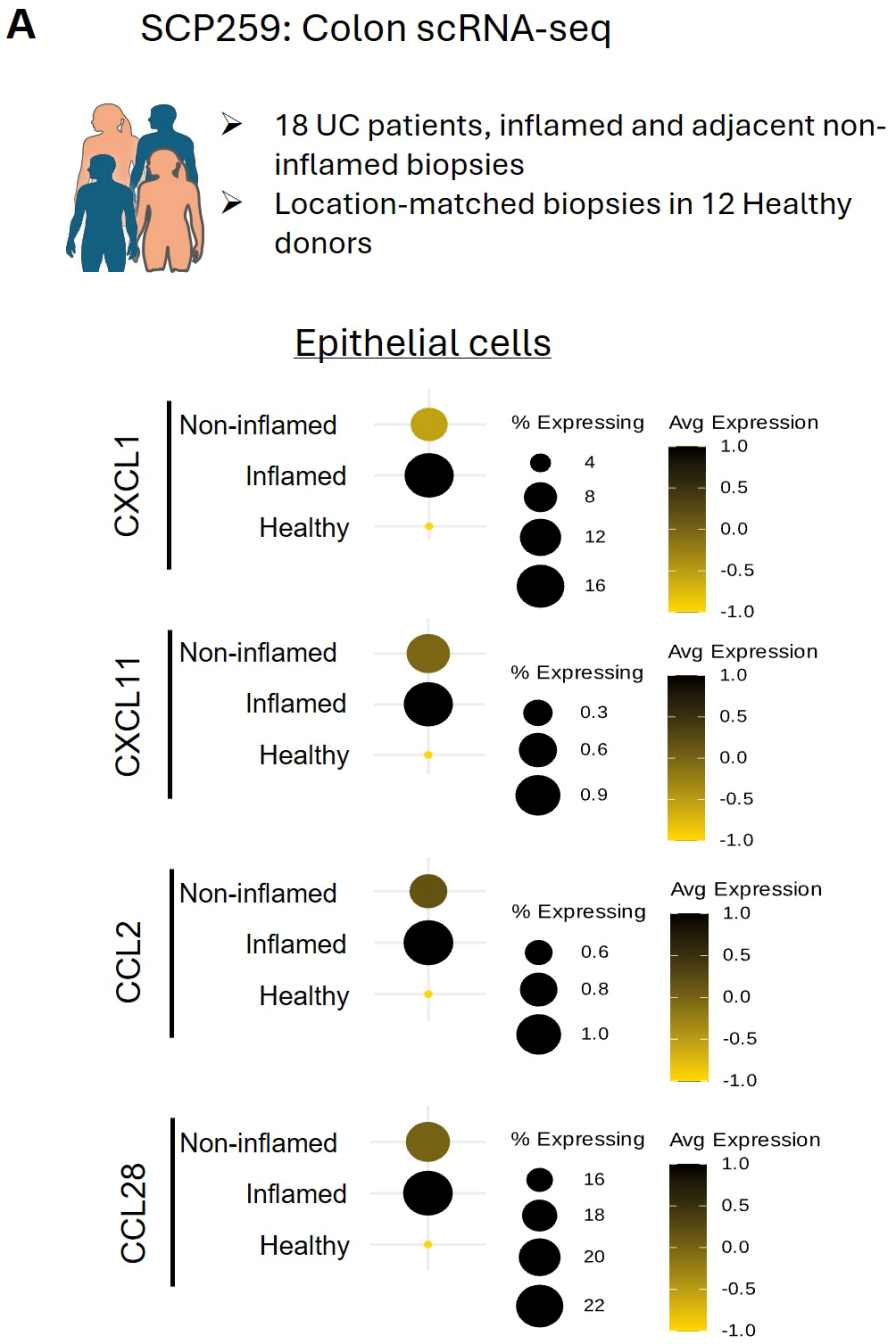
Epithelial-intrinsic chemokine expression in human UC colonoids and tissues. **(A)** Dot plots from the human colon scRNA-seq data (single cell portal accession=SCP259) showing chemokine expression in epithelial cells from non-inflamed UC, inflamed UC, and healthy donor biopsies.

To validate our previous findings of upregulated epithelial chemokine levels in UC compared to non-IBD control organoids (6), we explored a publicly available scRNA-seq dataset (13) containing paired inflamed and non-inflamed colonic biopsies from 18 UC patients and 12 healthy donors (**Figure 2**). Data from the epithelial cluster revealed a progressive increase in chemokine expression from healthy tissue to non-inflamed UC mucosa and, ultimately, to inflamed mucosa (**Figure 2**), suggesting that epithelial chemokine activation precedes overt histologic inflammation and may extend into regions that appear endoscopically quiescent.

**Figure 3:**
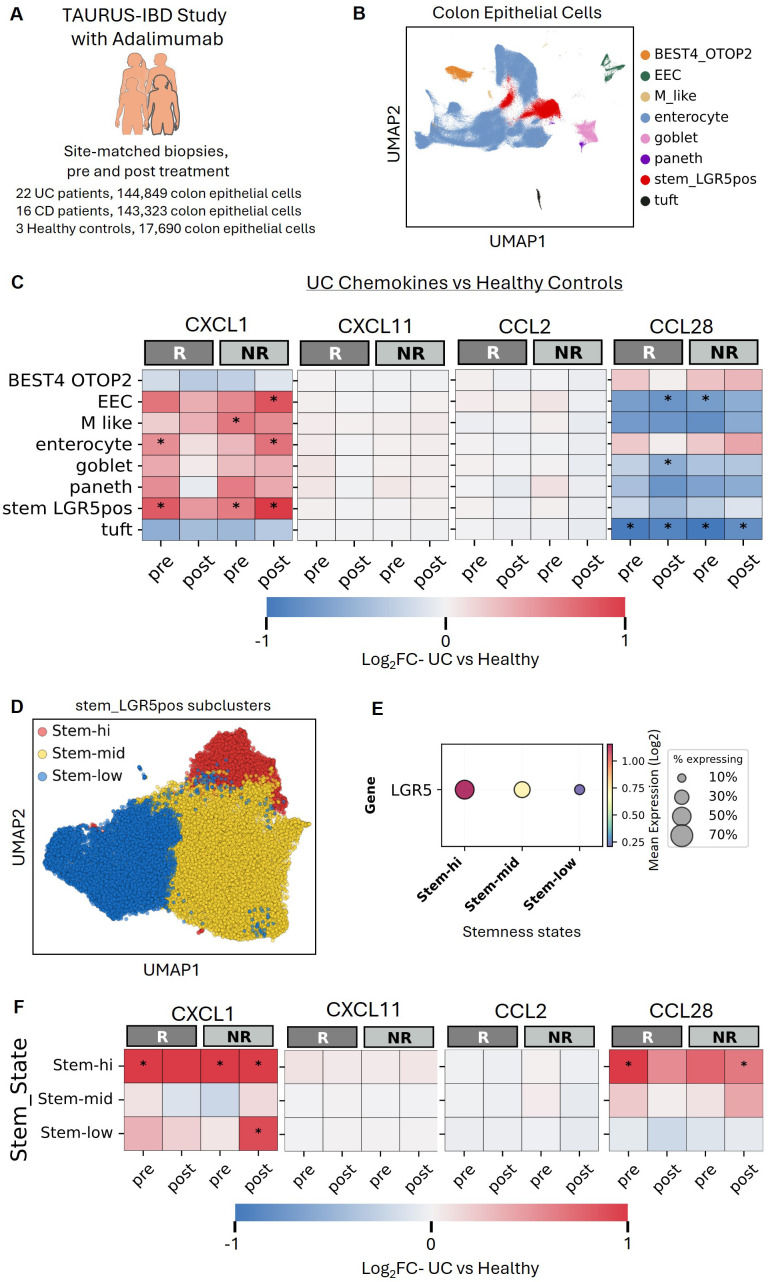
Stem cell and enterocytes derived CXCL1 track clinical remission in ulcerative colitis. **(A)** Schematic of the TAURUS-IBD study design. **(B)** Uniform Manifold Approximation and Projection (UMAP) of 305,862 colonic epithelial cells colored by cell type. **(C)** Heatmaps showing expression of chemokines relative to healthy controls across epithelial cell types in UC patients. **(D)** UMAP of 33568 LGR5-positive stem cells sub-clustered by stemness state. **(E)** Mean expression level of LGR5 in stem subclusters. **(F)** Heatmaps showing expression of chemokines relative to healthy controls across LGR5 stem cell subclusters in UC patients. In **(C, F)**, columns represent remission (R) and non-remission (NR) patients before (pre) and after (post) adalimumab treatment. Asterisks denote FDR-adjusted p < 0.01 and |Log_2_ FC| ≥ 0.5 compared to healthy controls.

**Figure 4:**
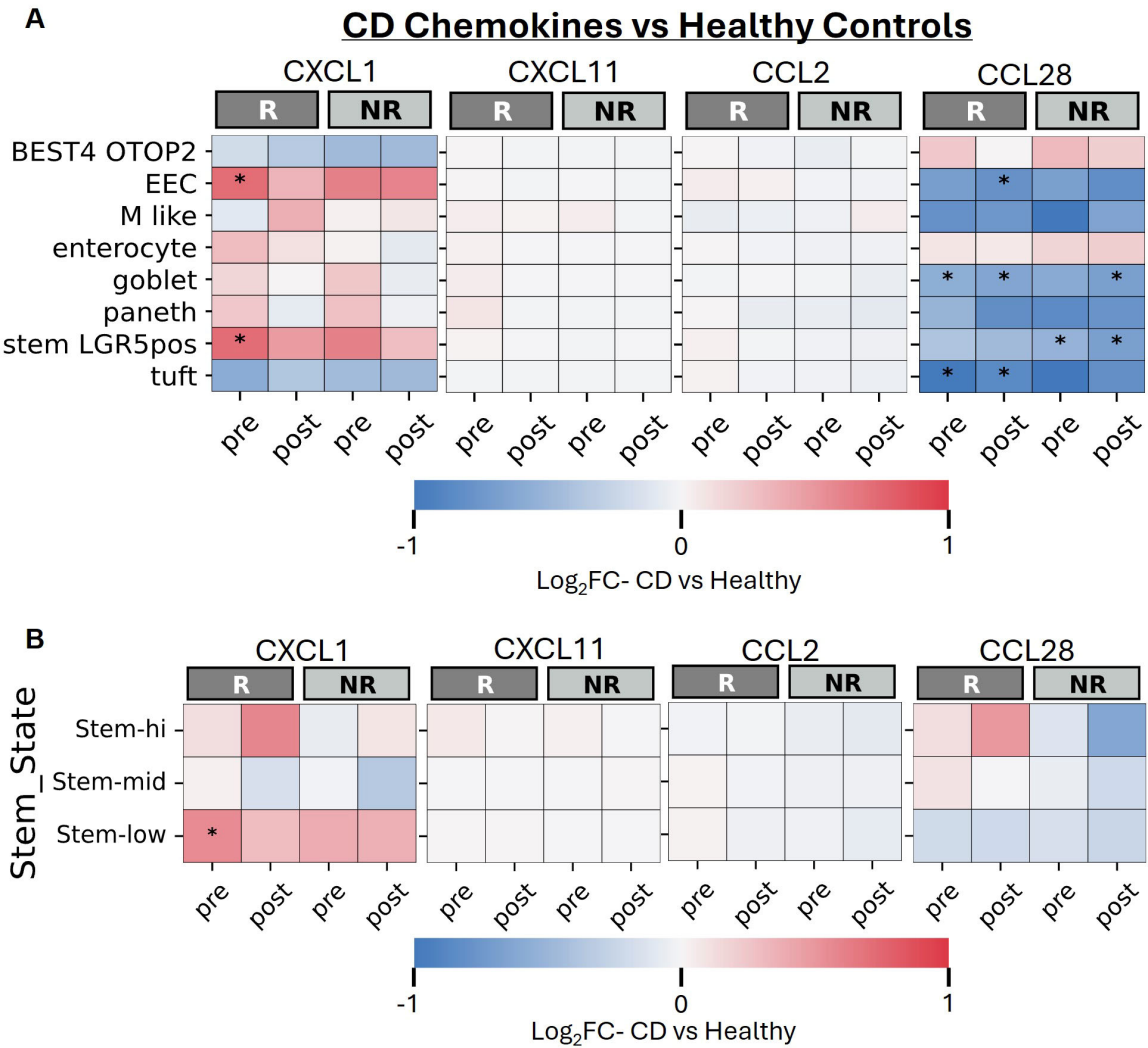
Crohn’s epithelial chemokines lack a predictive pattern in responders and non-responders. **(A)** Heatmaps showing expression of chemokines relative to healthy controls across epithelial cell types in CD patients. **(B)** Heatmaps showing expression of chemokines relative to healthy controls across LGR5 stem cell subclusters in CD patients. Columns represent remission (R) and non-remission (NR) patients before (pre) and after (post) adalimumab treatment. Asterisks denote FDR-adjusted p < 0.01 and |Log_2_ FC| ≥ 0.5 compared to healthy controls.

To identify the epithelial cellular sources underlying disease and remission patterns, we interrogated single-cell RNA sequencing data from the TAURUS-IBD study (9), encompassing over 305,862 colonic epithelial cells from UC and CD patients receiving adalimumab therapy (**Figures 3A, B**). In the UC data, only CXCL1 attained significant upregulation in enterocytes and organoid-forming LGR5-positive stem cells clusters (14) (**Figure 3C**). Importantly, clinical remission was associated with normalization of CXCL1 expression in stem cells and enterocytes, while non-remission patients maintained elevated expression after therapy (**Figure 3C**). Since patient-derived organoids are efficiently derived from adult stem cells (2, 14), we sought to obtain more insight into the chemokine profiles in the stem compartment in responders and non-responders, by stratifying cells in the stem compartment as high, mid, or low stemness states based on the LGR5 expression levels (**Figures 3D, E**, **Supplementary Figure 2**). CXCL1 and CCL28 were significantly expressed only in stem-hi state, and was not significant following therapy in responders, whereas CXCL11 and CCL2 did not exhibit any changes in expression across all stem cell states (**Figure 3F**). In contrast, patients with Crohn’s disease (CD) exhibited distinct patterns that are independent of clinical remission (**Figure 4A**). Following adalimumab treatment, the chemokines showed near-healthy levels in both remission and non-remission groups across most epithelial cell types (**Figure 4A**). Consistently, the chemokine expression pattern across stem cell states did not follow any clear pattern in the CD cohort (**Figure 4B**), suggesting that epithelial chemokine programs are uncoupled from clinical response in Crohn’s disease.

## Discussion

Chemokine-directed leukocyte trafficking to the sites of injury is a hallmark of inflammation in IBD (3). Recently, the overexpression of CXCL1, CXCL11, CCL2, and CCL28 was reported in colon epithelial organoids derived from UC patients (6). In this report, we used bulk and single-cell transcriptomic data from 4 clinical trials with anti-TNFα and anti-integrin therapies to investigate whether chemokines retained in UC epithelial organoids improved in patients who achieved clinical remission after treatment. Bulk transcriptomic analyses showed a consistent inverse relationship between epithelial chemokines, especially CXCL1 and CXCL11, and clinical remission in UC patients for all evaluated therapies (with CXCL1 showing strongest relationship). Similarly, epithelial chemokine normalization distinguished responders from non-responders through cell-type-specific resolution of CXCL1 in the enterocyte and LGR5 stem progenitor compartments in UC colon, but not in Crohn’s. These data demonstrate that CXCL1 retained in UC organoids could serve as biomarkers of treatment response in UC but not in CD, highlighting disease-specific differences that can be functionally targeted in organoid models.

In bulk analyses, CXCL1 showed the greatest reduction and predictive value after biologic therapy in UC. The stronger discriminatory performance of CXCL1 ROC analyses observed in the vedolizumab cohort compared with anti-TNF cohorts could be due to many reasons including differences in the duration of treatment. The vedolizumab cohort included longer-term follow-up samples (up to 52 weeks), potentially allowing greater divergence in CXCL1 expression between remission and non-remission groups compared with the post-treatment assessments available in the anti-TNF cohorts (both of which lasted up to 6 weeks).

Our analyses demonstrated that normalization of chemokine expression within enterocytes and progenitor compartments was associated with clinical remission. Considering their abundance, enterocytes are likely to contribute substantially to total chemokine output; highlighting the significance of chemokine resolution in this compartment. Likewise, the identification of similar dynamics in LGR5-positive stem cells is notable, given their role in organoid generation and epithelial renewal (14). Importantly, the presence of chemokine programs within progenitor compartments suggests that inflammatory signals may be encoded at the level of epithelial regeneration rather than confined to differentiated cells. The previously reported chemokine overexpression in active UC organoids is only prominent during differentiation (6), and agrees with the concept of inflammatory memory in the stem-cell compartment (15). Our findings in the stem-hi state are consistent with the possibility that epithelial progenitor states contribute to inflammatory programs in UC, although mechanistic studies will be required to determine whether modulation of these programs directly influences therapeutic response. Moreso, because only 27% of UC patients in the TAURUS-IBD study achieved remission (9), candidate therapies targeting CXCL1 disparities retained in patient-derived IBD epithelial organoids may increase the likelihood of CXCL1 normalization in stem cells, reduce leukocyte trafficking to the epithelium, and improve the odds of clinical remission in UC.

The divergence observed between UC and CD provides additional insight into disease-specific mechanisms, especially for epithelial-immune interactions. In UC, epithelial CXCL1 expression appears tightly coupled to clinical outcomes, implying a direct relationship between epithelial state and disease activity. In contrast, the normalization of CXCL1 in CD, regardless of clinical response, suggests that the epithelial pathway for this chemokine may be less central to disease persistence, or that other factors, such as immune compartment dynamics, may play a more dominant role. This distinction may have important implications for therapeutic development, as it suggests that epithelial-targeted strategies may differ in UC and CD. However, it is noteworthy that the disease-specific difference in epithelial CXCL1 could be due to sampling location, as it is plausible that samples from other intestinal locations in CD other than the colon may exhibit distinct epithelial chemokine signatures in remission. Alternative explanations also include differences in tissue architecture, anti-TNF responses in CD may involve cellular and molecular pathways that are less dependent on epithelial chemokine programs than those observed in UC, and potential differences in tissue sampling. Hence, the cohort composition may contribute to the observed divergence and should be evaluated in future studies.

This study has several limitations. First, all analyses were performed using publicly available transcriptomic datasets and should therefore be considered hypothesis-generating. Although the reproducibility of findings across multiple independent cohorts, therapeutic classes, and transcriptomic platforms strengthens confidence in the results, the scope of these analyses did not cover experimental validation. Hence, future studies should consider validating the relevance of epithelial CXCL1 to therapeutic response in IBD. Second, differences among cohorts, therapeutic regimens, disease severity, sample processing, and tissue sampling strategies may introduce residual confounding despite normalization and batch-correction procedures. Although LGR5-positive stem cells were robustly annotated, we cannot exclude the possibility that additional regenerative epithelial stem cell populations contribute to chemokine regulation during mucosal healing. Future clinical studies that generate epithelial-focused datasets may provide higher resolution for further insight into chemokine dynamics across distinct epithelial progenitor states.

Overall, our bulk and single cell transcriptomic analyses identify CXCL1 as the epithelial chemokine most consistently associated with clinical disease activity and remission in UC. Epithelial CXCL1 expression distinguishes responders from non-responders in UC but not in CD, suggesting disease-specific epithelial-immune interactions in the colon. The autonomous overexpression of CXCL1 in patient-derived UC organoids validates it as a functional readout for epithelial-directed therapeutic screening using patient-derived organoid models.

## Data Availability

The original contributions presented in the study are included in the article/**Supplementary Material**. Further inquiries can be directed to the corresponding author.
